# Supplementary material for: Unveiling the Bioactive Potential of the Invasive Jellyfish Phyllorhiza punctata Through Integrative Transcriptomic and Proteomic Analyses
Source: Biomolecules. 2025 Aug 4;15(8):1121. doi: 10.3390/biom15081121 (PMC12383608; doi:10.3390/biom15081121)
Supplement: Supplementary file 1 [file biomolecules-15-01121-s001.zip › Suplementary Material/FileS3/PEPFOLD4/XP_065067195_5075/clusters.html]

 PEP-FOLD clusters


Download archive of all models  here   

|  |  |  |
| --- | --- | --- |
| # | file | sOPEP |
| # cluster 1 - Size: 13 |  |  |
|  | XP\_065067195\_5075-model1 | -22.2856 |
|  | XP\_065067195\_5075-model1.1 | -20.6193 |
|  | XP\_065067195\_5075-model1.2 | -20.2406 |
|  | XP\_065067195\_5075-model1.3 | -19.5556 |
|  | XP\_065067195\_5075-model1.4 | -19.5556 |
|  | XP\_065067195\_5075-model1.5 | -19.4471 |
|  | XP\_065067195\_5075-model1.6 | -19.4279 |
|  | XP\_065067195\_5075-model1.7 | -19.4279 |
|  | XP\_065067195\_5075-model1.8 | -19.2815 |
|  | XP\_065067195\_5075-model1.9 | -19.2606 |
|  | XP\_065067195\_5075-model1.10 | -19.0079 |
|  | XP\_065067195\_5075-model1.11 | -18.0375 |
|  | XP\_065067195\_5075-model1.12 | -17.3032 |
| # cluster 2 - Size: 12 |  |  |
|  | XP\_065067195\_5075-model2 | -20.4858 |
|  | XP\_065067195\_5075-model2.1 | -20.4858 |
|  | XP\_065067195\_5075-model2.2 | -20.4858 |
|  | XP\_065067195\_5075-model2.3 | -19.0247 |
|  | XP\_065067195\_5075-model2.4 | -18.8333 |
|  | XP\_065067195\_5075-model2.5 | -18.8333 |
|  | XP\_065067195\_5075-model2.6 | -18.8333 |
|  | XP\_065067195\_5075-model2.7 | -18.8333 |
|  | XP\_065067195\_5075-model2.8 | -18.8333 |
|  | XP\_065067195\_5075-model2.9 | -17.2773 |
|  | XP\_065067195\_5075-model2.10 | -16.3403 |
|  | XP\_065067195\_5075-model2.11 | -15.3047 |
| # cluster 3 - Size: 5 |  |  |
|  | XP\_065067195\_5075-model3 | -20.1059 |
|  | XP\_065067195\_5075-model3.1 | -19.3094 |
|  | XP\_065067195\_5075-model3.2 | -16.2203 |
|  | XP\_065067195\_5075-model3.3 | -15.9513 |
|  | XP\_065067195\_5075-model3.4 | -14.6549 |
| # cluster 4 - Size: 4 |  |  |
|  | XP\_065067195\_5075-model4 | -19.6799 |
|  | XP\_065067195\_5075-model4.1 | -19.6788 |
|  | XP\_065067195\_5075-model4.2 | -19.6788 |
|  | XP\_065067195\_5075-model4.3 | -17.3416 |
| # cluster 5 - Size: 27 |  |  |
|  | XP\_065067195\_5075-model5 | -19.5761 |
|  | XP\_065067195\_5075-model5.1 | -19.5761 |
|  | XP\_065067195\_5075-model5.2 | -19.5761 |
|  | XP\_065067195\_5075-model5.3 | -19.5761 |
|  | XP\_065067195\_5075-model5.4 | -19.5761 |
|  | XP\_065067195\_5075-model5.5 | -19.5761 |
|  | XP\_065067195\_5075-model5.6 | -19.4827 |
|  | XP\_065067195\_5075-model5.7 | -19.4827 |
|  | XP\_065067195\_5075-model5.8 | -19.4827 |
|  | XP\_065067195\_5075-model5.9 | -19.4827 |
|  | XP\_065067195\_5075-model5.10 | -19.4757 |
|  | XP\_065067195\_5075-model5.11 | -19.4757 |
|  | XP\_065067195\_5075-model5.12 | -19.4757 |
|  | XP\_065067195\_5075-model5.13 | -19.4757 |
|  | XP\_065067195\_5075-model5.14 | -19.4757 |
|  | XP\_065067195\_5075-model5.15 | -19.4757 |
|  | XP\_065067195\_5075-model5.16 | -19.4757 |
|  | XP\_065067195\_5075-model5.17 | -19.4757 |
|  | XP\_065067195\_5075-model5.18 | -19.4757 |
|  | XP\_065067195\_5075-model5.19 | -19.4167 |
|  | XP\_065067195\_5075-model5.20 | -19.408 |
|  | XP\_065067195\_5075-model5.21 | -19.408 |
|  | XP\_065067195\_5075-model5.22 | -19.3786 |
|  | XP\_065067195\_5075-model5.23 | -19.3786 |
|  | XP\_065067195\_5075-model5.24 | -19.3521 |
|  | XP\_065067195\_5075-model5.25 | -19.3521 |
|  | XP\_065067195\_5075-model5.26 | -17.1681 |
| # cluster 6 - Size: 1 |  |  |
|  | XP\_065067195\_5075-model6 | -18.3296 |
| # cluster 7 - Size: 38 |  |  |
|  | XP\_065067195\_5075-model7 | -15.9339 |
|  | XP\_065067195\_5075-model7.1 | -15.8802 |
|  | XP\_065067195\_5075-model7.2 | -15.8429 |
|  | XP\_065067195\_5075-model7.3 | -15.8367 |
|  | XP\_065067195\_5075-model7.4 | -15.8236 |
|  | XP\_065067195\_5075-model7.5 | -15.7932 |
|  | XP\_065067195\_5075-model7.6 | -15.7932 |
|  | XP\_065067195\_5075-model7.7 | -15.7798 |
|  | XP\_065067195\_5075-model7.8 | -15.7374 |
|  | XP\_065067195\_5075-model7.9 | -15.7167 |
|  | XP\_065067195\_5075-model7.10 | -15.7107 |
|  | XP\_065067195\_5075-model7.11 | -15.7107 |
|  | XP\_065067195\_5075-model7.12 | -15.7001 |
|  | XP\_065067195\_5075-model7.13 | -15.7001 |
|  | XP\_065067195\_5075-model7.14 | -15.6843 |
|  | XP\_065067195\_5075-model7.15 | -15.6843 |
|  | XP\_065067195\_5075-model7.16 | -15.6838 |
|  | XP\_065067195\_5075-model7.17 | -15.6826 |
|  | XP\_065067195\_5075-model7.18 | -15.6776 |
|  | XP\_065067195\_5075-model7.19 | -15.5753 |
|  | XP\_065067195\_5075-model7.20 | -15.5657 |
|  | XP\_065067195\_5075-model7.21 | -15.4616 |
|  | XP\_065067195\_5075-model7.22 | -15.4258 |
|  | XP\_065067195\_5075-model7.23 | -15.4095 |
|  | XP\_065067195\_5075-model7.24 | -15.3549 |
|  | XP\_065067195\_5075-model7.25 | -15.247 |
|  | XP\_065067195\_5075-model7.26 | -15.247 |
|  | XP\_065067195\_5075-model7.27 | -15.1649 |
|  | XP\_065067195\_5075-model7.28 | -15.1518 |
|  | XP\_065067195\_5075-model7.29 | -15.1048 |
|  | XP\_065067195\_5075-model7.30 | -15.0632 |
|  | XP\_065067195\_5075-model7.31 | -14.9814 |
|  | XP\_065067195\_5075-model7.32 | -14.9694 |
|  | XP\_065067195\_5075-model7.33 | -14.9397 |
|  | XP\_065067195\_5075-model7.34 | -14.8542 |
|  | XP\_065067195\_5075-model7.35 | -14.8043 |
|  | XP\_065067195\_5075-model7.36 | -14.5622 |
|  | XP\_065067195\_5075-model7.37 | -13.9969 |
|  |  |  |
